# Thalamic Local Field Potentials Are Related to Long-Term DBS Effects in Tourette Syndrome

**DOI:** 10.3389/fneur.2021.578324

**Published:** 2021-02-15

**Authors:** Sara Marceglia, Marco Prenassi, Tommaso F. Galbiati, Mauro Porta, Edvin Zekaj, Alberto Priori, Domenico Servello

**Affiliations:** ^1^Dipartimento di Ingegneria e Architettura, Università degli Studi di Trieste, Trieste, Italy; ^2^Unità Operativa Neurofisiopatologia, Fondazione Istituto di Ricovero e Cura a Carattere Scientifico Ca'Granda Ospedale Maggiore Policlinico, Milan, Italy; ^3^Functional Neurosurgery Unit, Istituto di Ricovero e Cura a Carattere Scientifico Galeazzi Hospital, Milan, Italy; ^4^“Aldo Ravelli” Research Center for Neurotechnology and Experimental Brain Therapeutics, University of Milan Medical School, Milan, Italy

**Keywords:** Tourette syndrome, local field potentials, deep brain stimulation, tics, obsessive compulsive behaviors

## Abstract

**Background:** Local field potential (LFP) recordings helped to clarify the pathophysiology of Tourette syndrome (TS) and to define new strategies for deep brain stimulation (DBS) treatment for refractory TS, based on the delivery of stimulation in accordance with changes in the electrical activity of the DBS target area. However, there is little evidence on the relationship between LFP pattern and DBS outcomes in TS.

**Objective:** To investigate the relationship between LFP oscillations and DBS effects on tics and on obsessive compulsive behavior (OCB) comorbidities.

**Methods:** We retrospectively analyzed clinical data and LFP recordings from 17 patients treated with DBS of the centromedian-parafascicular/ventralis oralis (CM-Pf/VO) complex, and followed for more several years after DBS in the treating center. In these patients, LFPs were recorded either in the acute setting (3–5 days after DBS electrode implant) or in the chronic setting (during impulse generator replacement surgery). LFP oscillations were correlated with the Yale Global Tic Severity Scale (YGTSS) and the Yale–Brown Obsessive–Compulsive Scale (Y-BOCS) collected at baseline (before DBS surgery), 1 year after DBS, and at the last follow-up available.

**Results:** We found that, at baseline, in the acute setting, the power of the oscillations included in the 5–15-Hz band, previously identified as TS biomarker, is correlated with the pathophysiology of tics, being significantly correlated with total YGTSS before DBS (Spearman's ρ = 0.701, *p* = 0.011). The power in the 5–15-Hz band was also correlated with the improvement in Y-BOCS after 1 year of DBS (Spearman's ρ = −0.587, *p* = 0.045), thus suggesting a relationship with the DBS effects on OCB comorbidities.

**Conclusions:** Our observations confirm that the low-frequency (5–15-Hz) band is a significant biomarker of TS, being related to the severity of tics and, also to the long-term response on OCBs. This represents a step toward both the understanding of the mechanisms underlying DBS effects in TS and the development of adaptive DBS strategies.

## Introduction

Patients suffering from Tourette syndrome (TS) experience, often since the early childhood, a highly disabling condition characterized by motor and phonic tics, accompanied in most cases by behavioral and psychiatric comorbidities (1). While several TS patients benefit from pharmacological (2, 3) and behavioral (4, 5) therapies, there are others in which such treatments do not satisfactorily alleviate symptoms. In these refractory patients, deep brain stimulation (DBS) has emerged as alternative therapy, with promising results (6). In DBS, electrodes are positioned through stereotactic neurosurgery to a target structure and then connected to a subcutaneous pulse generator delivering the electrical therapy.

Several targets were proposed and tested for TS patients, with results showing a global efficacy of DBS over tics and psychiatric comorbidities, mainly obsessive–compulsive behaviors (OCB), which develops in several weeks or months (7). The specific anatomical target structure seems not to be a predictor of the efficacy, since DBS clinical effect depends on a combination of factors including clinical picture, patient's age, and comorbidities (8). The literature reports results of TS DBS targeted to the medial part of the thalamus (6, 9) [centro-median nucleus (CM), ventralis oralis nucleus (VO), parafascicular nucleus (PF)], the globus pallidus internus (9, 10) (GPi, anterior and posteroventrolateral parts), the internal capsule, the nucleus accumbens (9), the fields of Forel (H1) (11), and also the subthalamic nucleus (12).

DBS also provided the unique opportunity to record signals directly from the brain structures involved in TS pathophysiology, thus supporting both the study of the neurophysiological signatures of this complex pathology (13–17) and the improvement of DBS therapy *per se* (18–20). In particular, DBS electrodes allow recording the compound presynaptic and postsynaptic activity of neuronal populations in the area surrounding the lead, known as local field potentials (LFPs). LFPs are signals characterized by oscillations, mostly studied in the 2–45-Hz range. Following the large experience gained with DBS in Parkinson's disease, LFP analysis in TS led to interesting results, especially for the characterization of abnormal oscillations in the thalamus at rest (13, 14, 17, 19, 21), during tics (14, 17, 22), and during voluntary movements (17).

More specifically, TS LFPs at rest are characterized by activity in the low-frequency (2–7 Hz) and alpha (8–13 Hz) bands, which are often considered as a broad “theta” band (16, 20).

This “theta” power was shown to be correlated with tic severity, and especially with preoperative YGTSS scores (16), and was observed as a consistent signature of tic onset, but not of voluntary movement (17).

For this reason, the design and implementation of new “intelligent” DBS approaches, able to adopt stimulation parameters (23) and to deliver responsive stimulation automatically, are based on LFP recordings and, for TS, on this band (19, 20).

Despite this increasing knowledge, DBS in TS is known to be associated with postsurgery complications (6), leading to lead explant or to DBS switch OFF (24).

In this work, considering the LFP signal recorded in a cohort of patients followed for several years after thalamic DBS implant, we want to investigate whether and how LFP signatures are correlated with DBS outcomes and long-term follow-up.

## Methods

We retrospectively analyzed clinical data and LFP recordings from 17 patients (13 males; median age at DBS 31 years, range 20–52) with Tourette syndrome refractory to standard drug treatment, satisfying DSM-IV-TR (25) and World Health Organization criteria (26) treated and followed at the Functional Neurosurgery Unit in IRCCS Galeazzi Hospital (Milan) from November 2004 to December 2017. Table 1 reports the details of patients included in this retrospective analysis.

**Table 1:** Patients' clinical characteristics.

**Patient *N***	**Sex**	**Left/right dominance**	**Age at DBS**	**Education (years)**	**Last follow-up (years from DBS)**	**Years from DBS of chronic LFP**	**DBS state at last follow-up**	**LFP recording**
1	M	R	39	8	10	N/A	Removed	Acute
2	M	R	25	13	10	N/A	ON	Acute
3	M	R	24	8	10	N/A	Removed	Acute
4	M	R	52	8	10	N/A	Removed	Acute
5	F	R	47	13	9	N/A	ON	Acute
6	M	R	27	8	8	N/A	Removed	Acute
7	M	R	40	13	4	N/A	ON	Acute
8	M	R	25	13	13	N/A	Removed	Acute
9	F	S	29	13	11	N/A	ON	Acute
10	M	R	25	13	13	7	Removed	Chronic
11	M	R	33	13	12	5	ON	Chronic
12	M	R	46	8	4	2	ON	Acute and chronic
13	F	R	20	8	11	4	ON	Chronic
14	M	R	31	13	11	4	ON	Chronic
15	M	R	23	8	8	1	ON	Acute and chronic
16	F	R	45	13	11	4	ON	Chronic
17	M	R	48	13	10	N/A	Removed	Acute

All patients underwent neurosurgical procedures for the implant of DBS electrodes (model 3389 Medtronic, Minneapolis, USA) in the ventralis oralis/centromedian-parafascicular (Vo/CM-Pf) under general anesthesia, as detailed elsewhere (13). This intralaminar target differs from the classical target reported by Vandewalle, being shifted 2 mm anteriorly in order to more completely enclose the ventralis oralis nuclei into the stimulated area. Contact 0 was placed at the target (13). Years after, patients also underwent standard surgical procedure for the replacement of the implantable pulse generator (IPG), as discussed in Marceglia et al. (19).

Informed consent and Ethical Committee approval for data collection and analysis were obtained at the time of DBS surgery. As stated in the Informed Consent, all patients agreed to the recording procedure for research purposes. The procedure did not add any specific risk and did not change the clinical practice, being performed in the time window in which the leads are not connected to the IPG, as demonstrated by the large experience with Parkinson's disease.

### Data Collection and LFP Recordings

The Yale Global Tic Severity Scale (YGTSS) and the Yale–Brown Obsessive–Compulsive Scale (Y-BOCS) were collected at baseline (before DBS surgery), 1 year after DBS, and at the last follow-up available, as detailed in Table 2. In five patients, we also collected YGTSS and YBOCS at the time of IPG replacement.

**Table 2:** Patients' clinical assessments.

**Patient *N***	**YBOCS**				**YGTSS**			
	**Baseline**	**1 year after DBS**	**Battery replacement**	**Last follow-up**	**Baseline**	**1 year after DBS**	**Battery replacement**	**Last follow-up**
1	12	10	*N/A*	36	75	40	N/A	66
2	14	9	N/A	6	64	40	N/A	10
3	21	18	N/A	40	70	32	N/A	70
4	31	28	N/A	25	80	50	N/A	28
5	17	20	N/A	8	45	29	N/A	10
6	17	0	N/A	35	28	14	N/A	60
7	3	5	N/A	5	65	35	N/A	35
8	23	11	N/A	48	79	48	N/A	80
9	38	28	N/A	32	85	70	N/A	35
10	32	18	8	30	92	40	25	36
11	21	14	5	5	89	38	10	10
12	20	14	6	10	78	30	14	20
13	25	19	8	8	78	40	20	20
14	30	26	5	25	42	25	15	20
15	0	2	2	0	28	25	15	20
16	5	11	5	2	56	31	19	15
17	35	25	N/A	38	94	35	N/A	44

*YBOCS, Yale–Brown Obsessive–Compulsive Scale; YGTSS, Yale Global Tic Severity Scale*.

LFPs were recorded in two different settings: the “acute” setting, in which LFPs were recorded 3–5 days after surgery for DBS electrode placement, from externalized electrode extensions, and the “chronic” setting, in which LFPs were recorded during the surgical procedure for IPG replacement, from implanted extensions disconnected from the replaced IPG, before the connection to the new stimulation device. Ten patients were recorded in the acute setting, five patients in the chronic setting, and two patients in both settings (Table 1).

The details of the acute setting recordings were previously reported (13). In summary, LFPs were recorded at rest while the patient was comfortably laying in his/her bed. Signals were bipolarly captured from the contact pairs available in the 4-contact Medtronic 3389 electrode (01-12-23). Rest recordings lasted for a minimum of 60 to a maximum of 180 s. The Galileo BE Light EEG amplification system (EBNeuro SpA, Italy) was used for acquisition (1,024 sampling rate, 12-bit quantization with 5-V range, 2–500-Hz preamplification filtering).

Chronic recordings were performed in the surgical room, while the patient was awake, after the neurosurgeon removed the discharged IPG in local anesthesia. Recordings were performed from a single contact pair including the contact activated for stimulation using the FilterDBS technology (27). Signals were band-passed (5–45 Hz) and amplified, then digitized at a 500-Hz sampling frequency and 12-bit quantization with a 5-V range.

All signals were stored and then analyzed with the Matlab software (version R2016A, The Mathworks, Natik, MA, USA).

### LFP Analysis

After automatic tic rejection with an *ad hoc* matlab program, signals were further visually inspected and segments >30 s were selected for the analysis. For each patient in which more than one channel was available, LFPs recorded from the contact pair including the electrode activated for stimulation were chosen for the analysis.

LFPs were analyzed in the frequency domain. The power spectrum was obtained using the Welch's averaged, modified periodogram method with 1-Hz resolution (28), as previously described (13, 19). To allow comparison between patients, considering the large inter-patient variability and the differences introduced by the recording equipment, the power spectra were normalized using a simple linear min–max normalization (zero to one) between the maximum and minimum power spectra values of the 5–45-Hz band.

After normalization, the spectral power in the bands of interest was computed. We considered, as previously reported, the frequency bands of interest for TS, namely, the low-frequency, or theta (5–7 Hz), alpha (8–12 Hz), low-beta (13–19 Hz), and high-beta (20–35 Hz). In addition, according to the literature (16, 20), we also considered the broad 5–15-Hz band, as this was reported to be correlated with TS pathophysiology.

To estimate the personalized power in each band, we performed a peak detection, in order to define the highest point of spectral power, and considered as “personalized” band power the integral of the power spectrum in a band of ±1 Hz.

### Statistical Analysis

All statistical analyses were conducted using the Matlab software; using Linear Regression Analysis, linear correlation was conducted using the Spearman's coefficient, since the cohort considered in this study is small. To correlate LFP features with clinical scales (YGTSS and YBOCS), we selected the LFP coming from the hemisphere with higher power in the personalized 5–15-Hz frequency band. The power spectrum features were correlated with the absolute values of the YGTSS and YBOCS clinical scales of the three different assessment time periods (baseline, 1 year after DBS and the last follow-up available) and their differences (e.g., YGTSS relative after DBS = YGTSS after DBS – YGTSS baseline) to evaluate the evolution in time. Considering 2 bands, beta and 5–15 Hz, and three time points for each scale, Bonferroni corrections (29) were set at a threshold of 0.05/6; thus, *p* < 0.011 was the adjusted alpha *p* < 0.05 for correlation.

## Results

### Patients and Spectral Features

Patients' clinical characteristics are reported in Table 2. Median follow-up was 10 years (range: 4–13). Median YGTSS at baseline was 75 (range 28–94), that at 1 year after DBS was 35 (range 14–70), and that at the last follow-up was 28 (range 10–80). Median YBOCS at baseline was 21 (range 0–38), that at 1 year after DBS was 14 (range 0–28), that and at the last follow-up was 25 (range 0–48). However, considering the last follow-up scores separately for patients with DBS ON and with DBS implant removed, we observed a significant difference in both YBOCS (median score with DBS ON: 7, range 0–32; with DBS removed 36, range 25–48, Wilcoxon rank-sum test *p* < 0.0001) and YGTSS (median score with DBS ON: 20, range 10–35; with DBS removed 60, range 28–80, Wilcoxon rank-sum test *p* < 0.0001).

**Figure 1:**
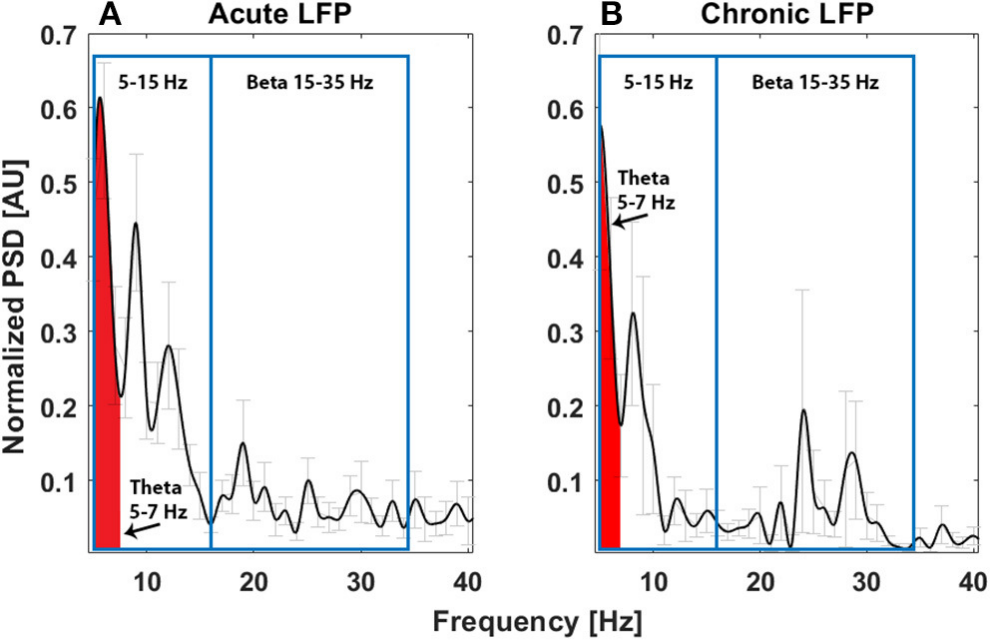
Average power spectrum in the acute (**A**, *N* = 11) and chronic (**B**, *N* = 6) experimental settings. The x-axis is the frequency (Hz), the y-axis is the normalized power spectral density (PSD, arbitrary unit), and light gray bars represent the standard error.

The power spectrum in the acute setting (Figure 1A) confirms the pattern reported for thalamic rest LFPs in TS (13, 16), characterized by a prominent activity in the low-frequency/theta and the alpha bands, as well as in the broad 5–15-Hz band. In fact, as shown in Figure 1A, averaging the power spectra across nuclei results in a multiple-peak pattern, broadening the low-frequency activity to the whole 5–15-Hz band.

**Figure 2:**
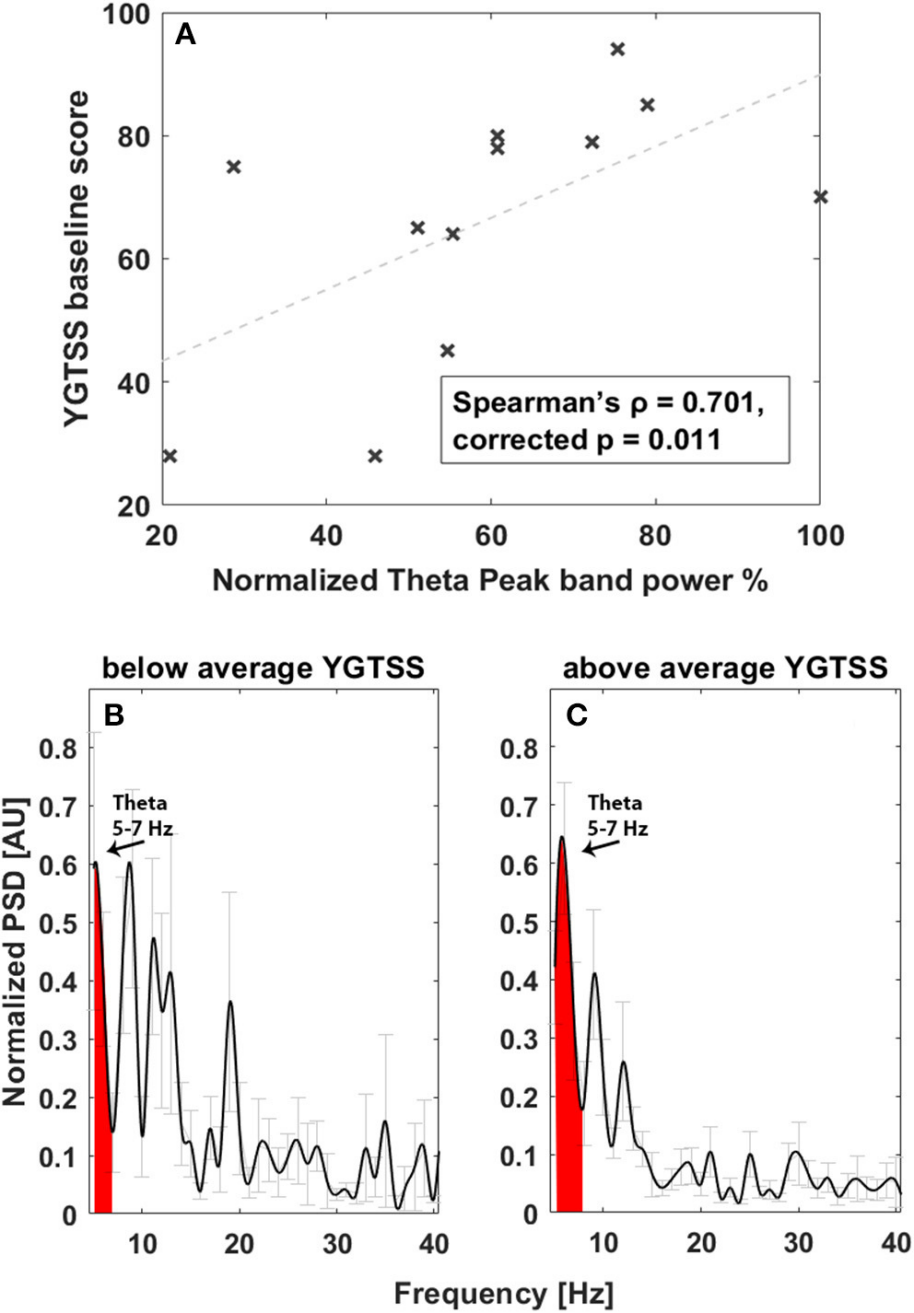
Correlation between tic severity and LFP power in the 5–7-Hz band at baseline. **(A)** Linear correlation results. The x-axis represents normalized theta (5–7 Hz) peak power; the y-axis represents the YGTSS baseline score. The dashed line represents the linear fit. Light gray bars represent the standard error. **(B)** Average power spectrum in the acute experimental settings in patients with YGTSS score below the average. The x-axis is the frequency (Hz), the y-axis is the normalized power spectral density (PSD, arbitrary unit), and light gray bars represent the standard error. **(C)** Average power spectrum in the acute experimental settings in patients with YGTSS score above the average. The x-axis is the frequency (Hz), the y-axis is the normalized power spectral density (PSD, arbitrary unit), and light gray bars represent the standard error.

Investigating the correlation between spectral feature and tics, considering the theta band in the 5–7-Hz range (Figures 2B,C), we found a significant correlation between total YGTSS scores at baseline and the spectral power of LFPs recorded in the acute setting, implying that a larger peak in this band is related to the intensity of tics (Spearman's ρ = 0.701, corrected *p* = 0.011, Figure 2A). No other correlations were found.

**Figure 3:**
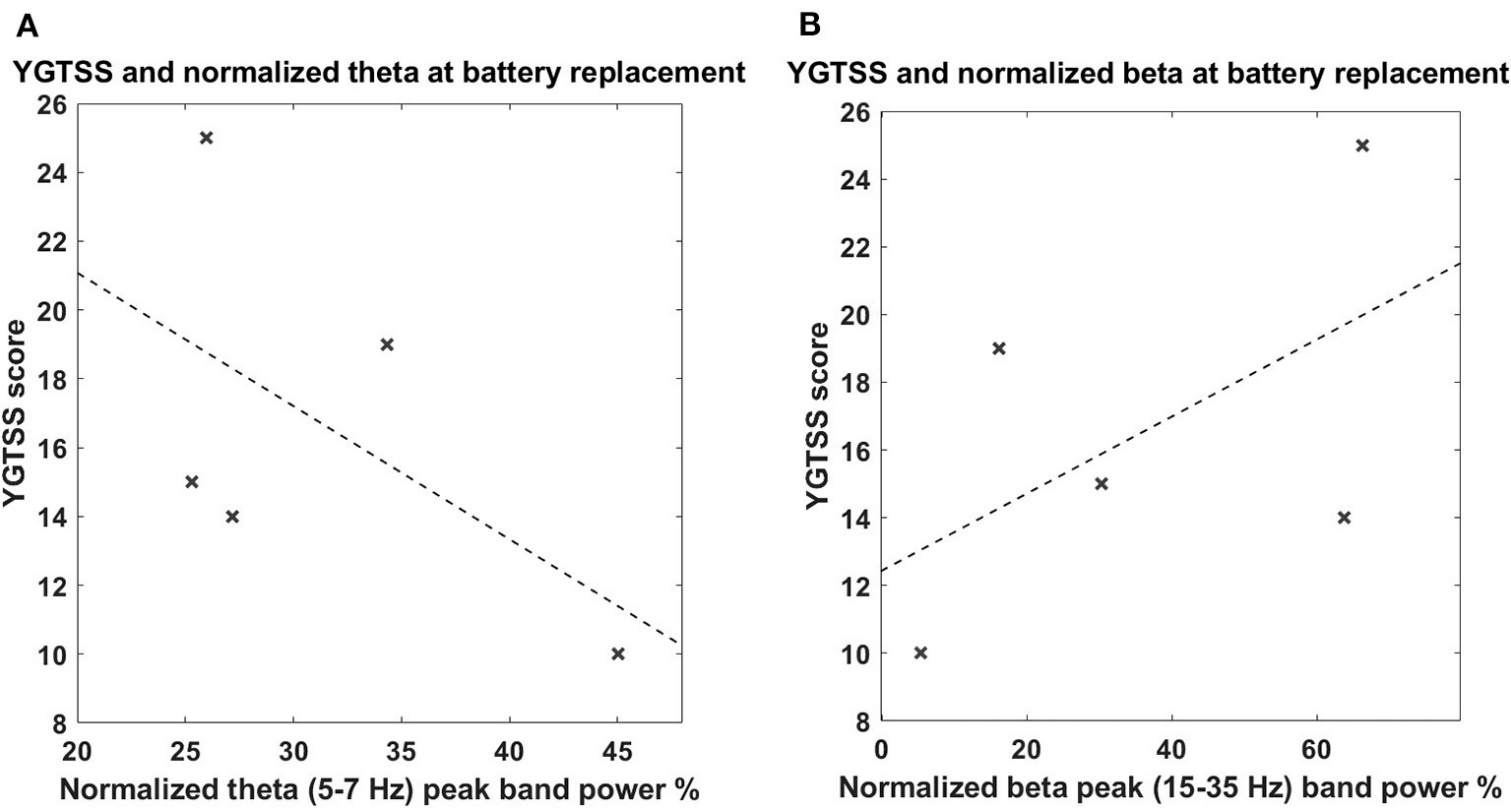
**(A)** Correlation between tic severity and LFP power in the 5–7-Hz band in the chronic setting. The x-axis represents the normalized theta (5–7 Hz) peak power; the y-axis represents the YGTSS score at the battery replacement. The dashed line represents the linear fit. **(B)** Correlation between tic severity and LFP power in the 13–35-Hz (beta) band in the chronic setting. The x-axis represents the normalized beta (13–35 Hz) peak power; the y-axis represent the YGTSS score at the battery replacement. The dashed line represents the linear fit. The correlations were not significant.

The power spectrum in the chronic setting (Figure 1B) shows a slightly different pattern: the activity in the broad 5–15-Hz band is more concentrated in the theta band, while a peak in the beta band arises from the average. We could not directly correlate the chronic LFPs with the YGTSS scores at battery replacement in all patients (five out of seven patients had the scores available), but, as shown in Figures 3A,B, there seems to be a negative correlation between beta power and YGTSS, in contrast with a possible positive correlation of the 5–15-Hz band power.

### Correlation Between LFP Spectral Features and DBS Effect

We then investigated whether spectral features characterizing the TS LFP pattern correlated with patients' clinical DBS outcomes. To do so, we considered the personalized frequency band in the 5–15-Hz range, which was mostly related to disease pathophysiology, and we considered the changes from baseline after 1 year of DBS and at the last follow-up.

Considering tics, we did not find any significant correlation between LFP features in the acute setting and YGTSS changes over time.

**Figure 4:**
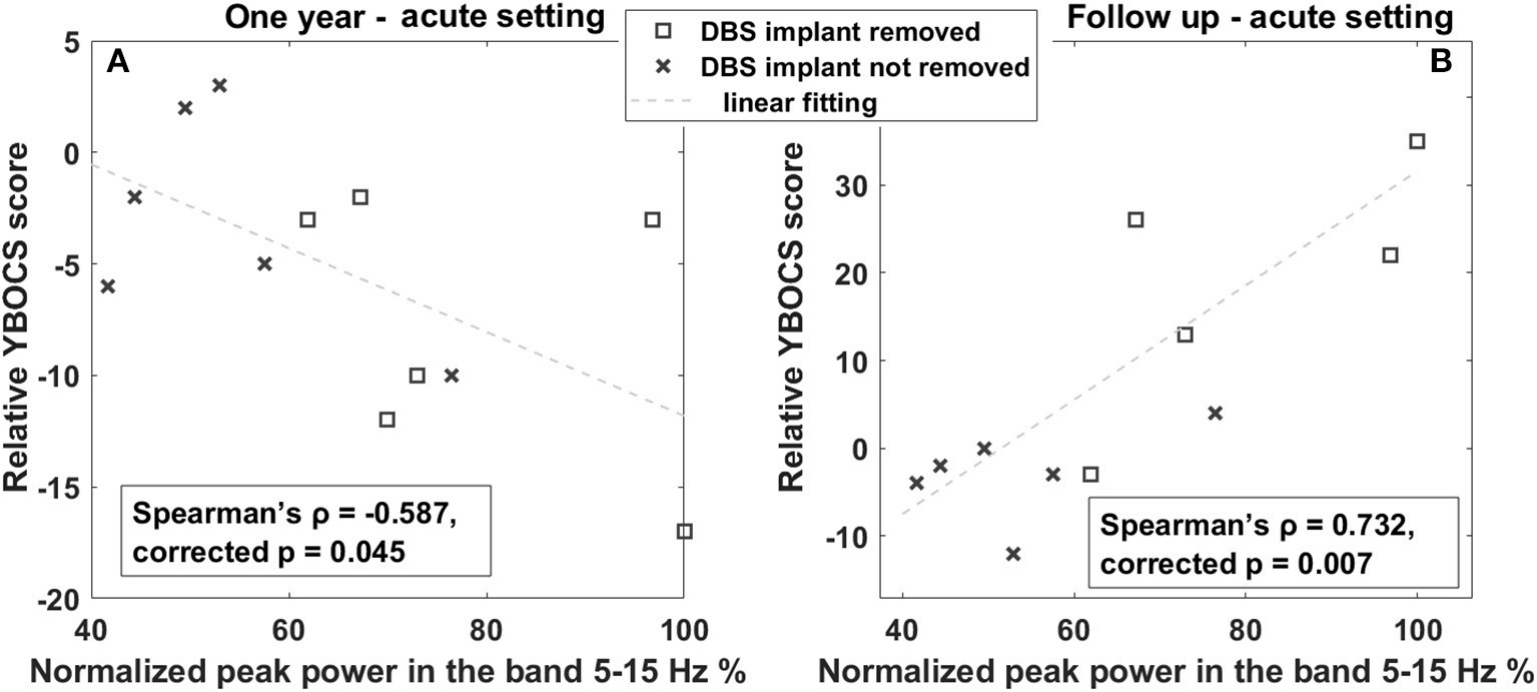
Correlation between OCB progression and LFP power in the 5–15-Hz band. **(A)** Linear correlation between YBOCS change 1 year after DBS and the power in the 5–15-Hz band measured in the acute setting. The x-axis represents the normalized peak power in the 5–15-Hz band expressed in percentage; the y-axis represents the difference between the YBOCS score assessed 1 year after DBS and the acute setting. The dashed line represents the linear fit. **(B)** Linear correlation between YBOCS change at the last follow-up and the power in the 5–15-Hz band measured in the acute setting. The x-axis represents the normalized peak power in the 5–15-Hz band expressed in percentage; the y-axis represents the difference between the YBOCS score assessed at the follow-up and in the acute setting. The dashed line represents the linear fit. Squares represent patients with DBS implant removed; crosses are patients with the DBS implant still implanted at the 1 year follow-up.

Conversely, considering OCB comorbidities, as shown in Figure 4, we observed an inverse correlation between YBOCS change 1 year after DBS and acute LFP spectral power at 5–15 Hz (Spearman's ρ = −0.587, corrected *p* = 0.045). This implies that a larger LFP spectral power at 5–15 Hz is associated with a larger improvement (decrease) in the YBOCS score. In line with this, investigating the correlation of the same band with the change at follow-up, where the clinical response was characterized by a general worsening, due to the fact that some patients were explanted, we found a direct correlation (Spearman's ρ = 0.732, corrected *p* = 0.007). In fact, in Figure 4B, the differentiation between patients with DBS implant (crosses) and those with DBS implant removed (squares) is clearly visible: patients still having a DBS implant are concentrated in the low-left corner of the plot, showing an improvement similar to that observable in Figure 4A; patients explanted are all worsening, and the worsening seems to be proportional to the amplitude of low-frequency oscillation. As an additional observation, and to rule out the possibility that larger low-frequency activity was predictive of DBS explant, we verified that there was no statistically significant difference in the low-frequency peak between patients still implanted at follow-up and those explanted (explanted LFP spectral power in the 5–15-Hz mean: 76.22 ± 16.52 vs. still implanted mean: 53.76 ± 12.50, *p* > 0.05, Wilcoxon rank-sum test).

**Figure 5:**
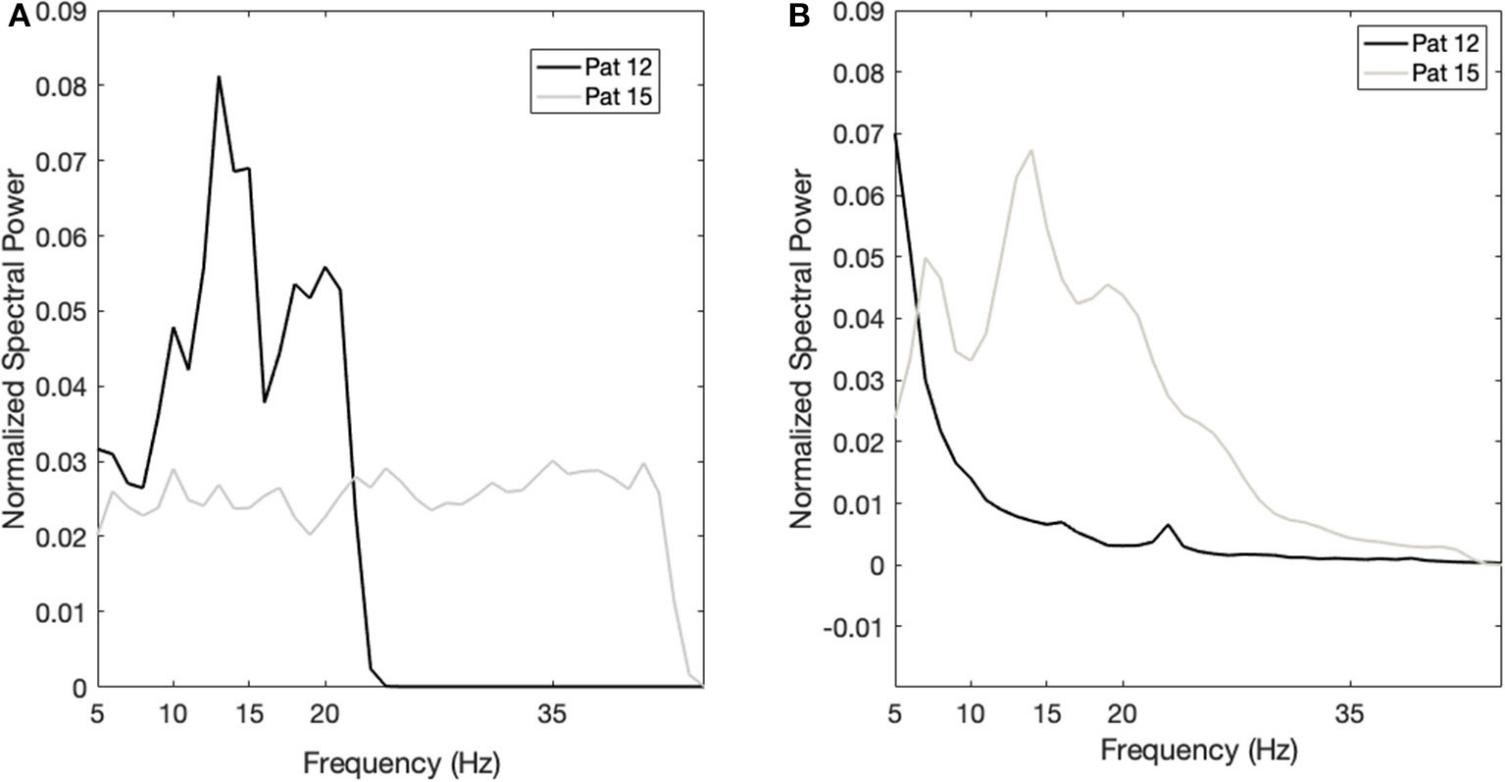
Case studies. **(A)** Power spectrum of patient 12 (black line) and patient 15 (gray line) in the acute setting. The x-axis is the frequency (Hz); the y-axis is the normalized power spectral density (PSD). **(B)** Power spectrum of patient 12 (black line) and patient 15 (gray line) in the chronic setting. The x-axis is the frequency (Hz); the y-axis is the normalized power spectral density (PSD).

To further corroborate these observations, we investigated the LFP changes in the two patients (patient n. 12 and n. 15), for which we have both acute and chronic recordings. Both patients are males and underwent bilateral Cm-Pf/VO DBS, one at the age of 23 (n. 15) and one at 46 (n. 12). At baseline, patient n. 12 had a larger YGTSS score (78 vs. 28), as well as a larger power in the 5–15-Hz band, whereas patient n. 15 had no defined peaks in this band. This larger 5–15-Hz power is also in line with the evolution of the OCB comorbidities: in fact, patient 12 showed a larger improvement in the YBOCS (−6 vs. +2) than patient 15 did, as observed in the general population. Figure 5 shows the power spectra of the two patients in the acute and chronic settings.

## Discussion

In this work, we investigated the relationship between thalamic LFP signatures in TS and the long-term effect of DBS and found that the spectral power in the 5–15-Hz band, previously identified as related to TS biomarker, is correlated with the pathophysiology of tics and with the DBS effects on OCB comorbidities. Also, we showed a progressive shift of the frequency band related to tics over time. We worked on a retrospective series of 17 patients that were followed for more than 10 years in one of the largest European centers for TS DBS.

Our work confirms previous findings on the thalamic LFP signatures of TS: as previously reported, we found a predominant activity in the lower portion of the frequency bands usually studied (namely, 2–45 Hz). More specifically, we identified the largest activity in the 5–15-Hz band, which includes the so-called low-frequency (or theta) band (2–7 Hz), the alpha band (8–12 Hz), and the slower portion of the low-beta band (13–20 Hz). This broad band was identified both in early studies from our group investigating thalamic LFPs (13) and in more recent studies investigating the relationship between tics and thalamic/pallidal LFPs (16). In addition, the same band was used in the first attempt to develop adaptive DBS for TS (20), in which stimulation was provided on demand according to the changes of spectral power in this broad band.

This activity is considered a general signature of the cortico-basal ganglia-thalamo-cortical loop related to hyperkinesia, being observed in other movement disorders such as dystonia (30, 31), or levodopa-induced dyskinesias (32, 33). This might explain the correlation observed between the power in this band and tic severity.

However, we found no correlation between tic severity improvement (YGTSS change over time) and theta power, nor did we observed a consistent decrease in this band over time while DBS was turned ON. Examining chronic LFPs, we however observed a shift of the peak in the 5–15-Hz toward lower frequencies (in the theta range), and a definition of a peak in the beta. This increase in the beta band is in line with previous findings on the effect of DBS on CM-LFPs. Maling et al. (34) reported an increased gamma (25–45 Hz) activity corresponding to DBS-related improvement. Their gamma activity is partially superimposed to our beta band, thus providing support to our findings, despite the different target. This may suggest that DBS could have induced a progressive shift toward higher frequencies of the tic signature. This is partially in line with the previous observation that, in chronic DBS patients, the alpha band is reduced when DBS is turned ON (19), but not lower frequencies. Another possible explanation is that DBS may induce an increase the power in very low frequencies (35), as observed in Parkinson's disease, in part due to polarization phenomena around the electrode, thus counterbalancing the effect of DBS on pathological low frequencies. However, both the alpha and the low-beta are in any case part of the broad 5–15-Hz band, and, therefore, this band may remain a consistent biomarker for adaptive DBS strategies. Also, frequency shifts within the same oscillatory band were shown to be representative of information encoding for movement at the basal ganglia level in movement disorders (36), thus confirming that the frequency modulations are a common mechanisms for brain signals.

Another interesting point is the correlation between theta power and OCB comorbidities, which is consistent over time: larger theta power before DBS is turned ON (as in the acute setting) is associated with larger improvement in YBOCS, thus suggesting to be a predictor of better thalamic DBS outcome on psychiatric comorbidities. Theta power in the basal-ganglia-thalamo-cortical loop has been shown to be related to nonmotor functions, such as decision processes (37), reward, and moral and ethical behaviors (38). For instance, larger responses in the theta power have been associated with compulsive behaviors in gamblers with Parkinson's disease (37). We can therefore hypothesize that DBS delivered in the Cm-Pf/VO target is able to interfere also with OCB comorbidities, especially in those patients with larger theta band before DBS is turned ON, possibly related to a positioning of the target electrodes in areas encoding also non-motor information. This observation is consistent with the fact that the Cm-Pf/VO targeted in our patients was chosen as 2 mm anterior as compared to the other well-known thalamic target (39), in order to include more the associative part of the VO nucleus. These data may suggest that this target may be useful, for OCB treatment, in patients with a pattern of high low-frequency activity, but not for others characterized by a different LFP pattern. In fact, in some cases, it has been shown that a second “rescue” surgery was needed to address OCB comorbidities (40). However, even though the relationship between the target area, non-motor functions, and low frequencies can explain why DBS delivered to this area provides a good control of OCB comorbidities, the absence of a pre-DBS correlation between YBOCS and low-frequency power remains unclear. It is possible that the pre-DBS abnormally increased low-frequency oscillation, observable in different brain structures and pathologies such as in parkinsonian dyskinesias, dystonia, and pathological aggression (41, 42), could be related not only to motor disinhibition and motor overflow (21) but also to nonmotor behaviors, thus masking the correlation with YBOCS. Interestingly, as shown here and in other papers (21), years after DBS the pattern of low-frequency activity remains mostly unchanged, though more concentrated in the theta band, consistent with the improvement in YBOCS. These hypotheses are however speculative, and further research is needed to clarify these aspects.

All our present observations have implications for the development of adaptive DBS for TS. First, our results confirm that the 5–15-Hz is a significant biomarker of tics. However, we also observed that this band is characterized by a large intersubject variability, since different patients show the main activity at different frequencies. This implies that any adaptive DBS strategy should rely on the identification of personalized peaks of spectral activity that should be followed to drive automatic parameter changes. Second, we showed that there may be a progressive shift of the main frequencies related to tics in time, after years of DBS application. This implies that any adaptive DBS algorithm for Tourette should reconsider the definition of the personalized LFP signature over time, adapting not only the stimulation but the algorithm *per se*. Third, we showed that the 5–15-Hz signature could be predictive of patients' outcome for OCB comorbidities. This implies that adaptive DBS could be designed for not only motor TS symptoms but also other comorbid symptoms, such as OCBs, thanks to the investigation of specific LFP oscillations.

This work was limited by its retrospective nature. We could analyze only patients that were recorded at the time of DBS implant, which did not correspond, in most cases, to those recorded at IPG replacement. This did not allow to define a clear intrasubject evolution of the LFP pattern, which would have strengthened our results. However, the two cases here shown for which all data and recordings are available support the results at the population level, especially for the relationship between the 5–15-Hz band and DBS outcomes. Also, the number of subjects with LFP recordings (17 patients) is much lower than the full series of cases treated at the Galeazzi hospital. Therefore, our results suggest that a systematic collection of LFP data, even in simple experimental conditions (baseline, rest recordings), could open the way to large improvements in DBS therapy. Another limitation relates to the fact that some patients were explanted or switched off at the time of follow-up, thus making it difficult to fully interpret the correlations observed between baseline LFPs and long-term outcomes.

In conclusion, our results show that LFPs in the broad 5–15-Hz range are representative of the tic severity at baseline and may be related to better outcomes of thalamic DBS on OCB comorbidities. In addition, in time, the LFP pattern, despite remaining mostly concentrated in the same frequency band, shows a progressive frequency shift, moving the oscillation related to tics toward the faster portion of this broad band, up to the beta rhythm. These observations represent a step toward both the understanding of the mechanisms underlying DBS effects in TS and the development of adaptive DBS strategies.

## Data Availability Statement

The raw data supporting the conclusions of this article will be made available by the authors, without undue reservation.

## Ethics Statement

The studies involving human participants were reviewed and approved by IRCCS Istituto Ortopedico Galeazzi. The patients/participants provided their written informed consent to participate in this study. Written informed consent was obtained from the individual(s) for the publication of any potentially identifiable images or data included in this article.

## Author Contributions

SM and MPr: conceptualization, investigation, formal analysis, supervision, data curation, writing—original draft, and writing— review and editing. TG: investigation, writing—original draft, and writing—review and editing. MPo and EZ: investigation. AP: investigation and writing—review and editing. DS: conceptualization, investigation, and writing—review and editing. All authors contributed to the article and approved the submitted version.

## Conflict of Interest

SM and AP are founder and shareholder of Newronika Srl, a spin-off company of the Fondazione IRCCS Ca' Granda Ospedale Maggiore Policlinico and of the University of Milan. The remaining authors declare that the research was conducted in the absence of any commercial or financial relationships that could be construed as a potential conflict of interest. The reviewer VV-V declared a past co-authorship with two of the authors MPo and DS to the handling editor.

## Abbreviations

DBS: Deep Brain Stimulation
LFP: Local Field Potentials
OCB: Obsessive Compulsive Behaviors
TS: Tourette Syndrome
YBOCS: Yale-Brown Obsessive-Compulsive Scale
YGTSS: Yale Global Tic Severity Scale.
